# From Prototype to Clinical Workflow: Co-Developing a Wearable Hypomimia System for Parkinson’s Disease

**DOI:** 10.3390/s26144454

**Published:** 2026-07-14

**Authors:** Alexander Johannes Wiederhold, Lea Haidar, Monika Pötter-Nerger, Christopher Gundler

**Affiliations:** 1 Institute for Applied Medical Informatics, University Medical Center Hamburg-Eppendorf, Martinistr. 52, 20246 Hamburg, Germany; 2 Department of Neurology, University Medical Center Hamburg-Eppendorf, Martinistr. 52, 20246 Hamburg, Germany

**Keywords:** Parkinson’s disease, hypomimia, wearable electronic devices, digital health, usability, medical symptom assessment, iterative development, facial movement analysis, mHealth, System Usability Scale

## Abstract

**Highlights:**

**What are the main findings?**

- A wearable, cap-mounted system for hypomimia capture was iteratively developed and successfully integrated into routine inpatient ON/OFF assessments in Parkinson’s disease.
- Redesign based on patient feedback improved usability, operationalized through the System Usability Scale, from moderate to good while preserving stable acquisition performance in clinical use.

**What are the implications of the main findings?**

- The findings suggest that engineering feasibility, workflow integration, and patient acceptability should all be considered when developing wearable facial monitoring systems for clinical use.
- This system provides a practical platform for future validation of digital facial markers in Parkinson’s disease.

**Abstract:**

Hypomimia, the reduced facial expressiveness commonly seen in Parkinson’s disease (PD), is clinically relevant, yet routine assessment usually relies on a single ordinal rating item in major rating scales such as the Unified Parkinson’s Disease Rating Scale (MDS-UPDRS). This study aimed to develop and iteratively refine a wearable, cap-mounted device for hypomimia recording during routine inpatient ON/OFF assessments and to examine its usability and technical feasibility. A staged co-development process with four prototypes was conducted. After clinician workflow requirements had been incorporated, Prototype 3 was evaluated during clinical routine using the System Usability Scale (SUS) and short free-text feedback. These findings informed a patient-centered redesign that resulted in Prototype 4. Bench testing confirmed stable operation, robust housing, and reliable closed Wi-Fi streaming. Usability improved from moderate in Prototype 3 (mean SUS 44.7, SD 18.2; *n* = 15) to good in Prototype 4 (mean SUS 80.0, SD 12.6; *n* = 50). Mixed-effects regression showed an increase of approximately 33 SUS points for the redesigned device (95% CI 27.7–39.4; *p* < 0.001). Mean effective sampling rate was 4.79 frames/s (SD 0.33), with face detection in 95.4% of frames. These feasibility and usability results indicate that technical robustness and patient-centered design are critical prerequisites for later clinical validation and support the integration of a wearable hypomimia capture device into routine assessments.

## 1. Introduction

Parkinson’s disease (PD) is the second most common neurodegenerative disorder of adult age and one of the leading causes of disability worldwide [1,2]. Over the past three decades, the global prevalence of PD has more than tripled, and recent estimates suggest that approximately 11.8 million people were living with PD in 2021, accompanied by substantial increases in age-standardized prevalence [2]. Clinically, PD is defined by the motor symptoms bradykinesia and at least rigidity and/or resting tremor [3] and is associated with a wide range of non-motor symptoms [1]. While these clinical manifestations are crucial for diagnosis and treatment decisions, they are typically assessed during brief, infrequent clinic visits. This approach may not fully capture the day-to-day variability of motor function and treatment response in real-world settings [4].

Among the motor manifestations of PD is hypomimia, characterized by reduced facial expressiveness and diminished facial movements, affecting up to 70–90% of people with Parkinson’s over the course of the disease [1,5]. Hypomimia is considered one of the early motor signs of PD and may precede classical limb motor features by several years, which renders it a potentially valuable marker for earlier detection and phenotyping [5,6]. Beyond its motor dimension, facial expressivity is tightly linked to social interaction, emotional communication, and interpersonal functioning. Reduced facial expression has been associated with altered emotion recognition and processing, social withdrawal, and reduced quality of life and can thus be considered as a non-motor symptom as well. Nevertheless, despite its clinical and psychosocial relevance, hypomimia remains comparatively under-studied and under-measured relative to limb motor symptoms, in part because simple and objective tools for its continuous assessment are lacking [1,5].

In routine clinical practice, facial expressivity is typically assessed using a single item (3.2 “facial expression”) of a standardized neurological rating scale, the Unified Parkinson’s Disease Rating Scale (MDS-UPDRS), rated on a 0–4 ordinal scale [7]. This approach is inherently subjective, depends on expert raters, and compresses complex, dynamic facial behavior into one coarse rating, limiting sensitivity to subtle, short-term, or context-dependent changes [1,5,6]. Standard neurological examinations and video-based ratings are usually performed at isolated time points in the clinic, which means that temporal fluctuations of hypomimia across medication cycles, emotional states, fatigue levels, and social situations remain largely unobserved [4,8]. As a consequence, clinicians may underestimate the real-world impact and variability of facial masking in everyday life.

In recent years, digital health approaches have introduced automatic, video-based analysis of facial movements in PD, demonstrating robust discrimination between individuals with PD and controls and enabling quantification of hypomimia from short, stationary recordings under controlled laboratory or clinic conditions [5,9]. However, hypomimia is inherently state- and context-dependent, as facial expressivity fluctuates with dopaminergic medication status, emotional and social context, and physical or mental fatigue. Consequently, brief clinic-based or laboratory recordings can only capture a narrow temporal window of the symptom and may fail to reflect its real-world dynamics [1,5,9]. Addressing this gap requires monitoring solutions that are not only technically accurate, but also wearable, tolerable, and compatible with routine clinical workflows over extended periods.

Despite this need, many PD-focused digital health studies specify devices and study protocols in advance, involving clinicians and people with PD primarily as study participants rather than as active contributors to the development process [10]. User feedback is often collected only after data acquisition, typically at the end of participation, which limits the opportunity for iterative refinement based on real-world use [11,12,13]. This approach risks producing technically sophisticated systems that lack long-term acceptability, clinical feasibility, or relevance to patient priorities [14]. In contrast, early and continuous involvement of both clinicians and people with PD enables identification of practical constraints, usability barriers, and contextual factors that directly shape sustained use [15,16]. Co-developing monitoring technologies from the earliest clinically deployable prototypes, therefore, represents a critical step toward successful translation from research settings into routine clinical care [17,18].

The objectives of this study are threefold. First, we develop a novel, mobile hypomimia capture system in close collaboration with movement disorder clinicians and people with PD, integrating a camera system into a 3D-printed hardcase that closely monitors facial landmarks. Second, we systematically evaluate the usability and acceptability of this device in a real clinical environment using the System Usability Scale (SUS) and short free-text feedback from patients, complemented by structured qualitative input from clinicians. Third, we use patient usability data and structured qualitative clinician input to guide iterative refinement of the device and app and to quantify usability changes between prototype versions. This development and feasibility evaluation focuses on acquisition stability, workflow compatibility, SUS-based usability, and redesign-relevant patient and clinician feedback. Together, these endpoints position the final wearable, mHealth-compatible system as a practical platform for subsequent validation of digital hypomimia markers in clinical and research applications.

## 2. Materials and Methods

**Figure 1:**
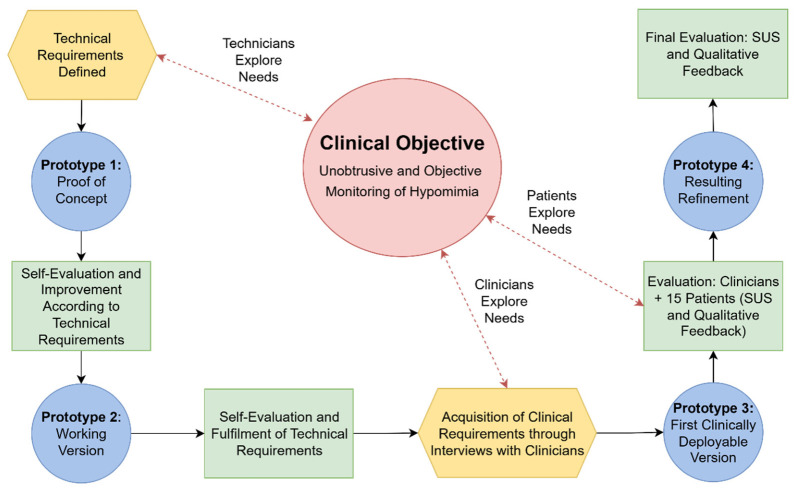
Flowchart showing the iterative development process of our hypomimia capturing device.

To achieve the objectives outlined above, we adopted a staged, iterative development and evaluation approach that integrates technical feasibility testing with clinical usability assessment. Accordingly, system development proceeded in two main phases. In a first, pre-clinical phase, early prototypes were developed and evaluated exclusively by technical experts and movement disorder clinicians to establish basic feasibility, safety, and suitability for clinical deployment. In a second phase, once a clinically deployable baseline configuration had been reached, the system was iteratively refined based on patient usability feedback and structured qualitative clinician input during routine inpatient assessments (Figure 1).

### 2.1. Technical Requirements

Engineering requirements were defined at the Institute for Applied Medical Informatics at the University Medical Center Hamburg-Eppendorf to ensure that the system can technically fulfill the clinical requirements while producing data suitable for later quantitative analysis.

First, the battery system should be rechargeable, easy to operate for clinical staff and, in future, for patients. The battery should operate without noticeable heat generation and ideally, batteries are exchangeable and support straightforward charging routines.

Although not strictly necessary for quantifying hypomimia, we considered an inertial measurement unit (IMU) a desirable extension. An IMU provides head-movement and posture information that can contextualize facial expressions and may enable additional applications such as fall detection or characterization of axial motor symptoms while the device is worn.

To integrate camera and IMU data, the processing unit must be powerful enough to retrieve and pre-process video and sensor streams, combine IMU readings with each video frame, and offer an integrated Wi-Fi module for direct communication with a mobile device. At the same time, it must remain energy-efficient, capable of running custom firmware, and should not generate excessive heat.

All hardware components must be embedded in a lightweight, robust housing that can be mounted on the head. To support rapid iteration of shapes and mounting concepts, a 3D-printable plastic enclosure would be favored that allows custom-fitted inlets for the components. The housing should prevent components from moving or loosening during everyday head movements and be accessible via standard screws to facilitate assembly, repair, and exchange of parts during early development phases.

### 2.2. Design Rationale

Building on the defined technical requirements, we explored wearable form factors capable of maintaining a stable camera position relative to the face while remaining acceptable for people with PD and practical for use in routine clinical settings. Together with clinicians, a range of body-worn concepts was discussed and sketched, including glasses-like devices, headbands, forehead or temple mounts, ear-hook cameras, and chest or collar-mounted solutions.

Each concept was evaluated against core constraints derived from the technical requirements, namely reliable full-face coverage, stability during head movements, unobtrusiveness, hygiene compatibility, and suitability for potential use beyond the hospital setting.

Chest- or collar-mounted cameras were deprioritized, as head movements frequently shift the face out of frame or into oblique perspectives. Glasses-based, headband, and ear- or temple-mounted configurations would, in principle, offer greater stability but were considered suboptimal for consistently capturing the entire facial region within a single frame. These considerations led to the selection of a head-mounted approach that anchors the camera on an everyday object already familiar to patients. A standard baseball cap emerged as a suitable carrier, as its brim sits at a reproducible distance in front of the face without obstructing the wearer’s field of view. The brim provides a mechanically stable platform for a compact camera housing while leaving the face largely accessible for neurological examination and routine clinical procedures.

### 2.3. Iterative Proof-of-Concept Development

Device development followed an iterative, user-centered process comprising two successive 3D-printed hardware prototypes, which were followed by another two clinically refined versions. All enclosures were produced using 3D printing to enable rapid adjustment of geometry, dimensions, and mounting concepts at low material cost. In the early development stages, prototypes were evaluated exclusively with clinicians and technical experts to establish technical proof-of-concept and safety prior to any patient exposure. Only once a technically stable and clinically deployable baseline had been reached were patients involved in systematic usability evaluation and co-development, as described in the Results Section 3.

Prototype 1 was implemented as a rectangular hardcase mounted on top of the cap brim using a Velcro fastener. The enclosure contained an ESP32-based processing unit connected to a wide-angle camera via a cable routed to the underside of the brim. Image frames were stored locally on a microSD card at a low sampling rate. In practice, this configuration did not allow reliable capture of the entire face within a single frame, as either upper or lower facial regions predominated depending on cap position. No live video preview was available, which meant that camera alignment could only be assessed retrospectively. This prototype was evaluated in bench tests and informal sessions with clinicians only. Feedback at this stage highlighted the need for a more brim-conformal geometry, improved stability of full-face coverage, and a design more compatible with clinical hygiene requirements.

Prototype 2 addressed these limitations by redesigning the enclosure as a negative form of the cap brim that could be slipped onto standard baseball caps, creating a more integrated unit. The 3D-printed structure incorporated a hollow core accommodating a custom printed circuit board with a camera connector, an ESP32-mini processing module, and an inertial measurement unit. The brim was extended beyond the textile edge to position the wide-angle camera at the distal tip, enabling full-face capture in test recordings. Custom firmware streamed video data over a dedicated Wi-Fi connection, allowing live video preview and precise adjustment of the cap position on the wearer. Although this prototype achieved stable full-face capture and real-time monitoring, it remained relatively heavy and depended on a nearby computer for operation. As with Prototype 1, evaluation was limited to clinicians and technical experts, whose feedback informed further refinement toward a configuration suitable for routine clinical deployment.

### 2.4. Transition to Clinical Co-Development and Usability-Based Iteration

The first two prototypes established a technical proof-of-concept for stable, head-mounted full-face video capture and real-time monitoring under controlled conditions. At this stage, development was deliberately limited to clinicians and technical experts to ensure safety, feasibility, and basic suitability for clinical environments before involving patients.

Building on this technical baseline, the subsequent development phase was designed to systematically integrate clinical and patient perspectives into the device refinement process. To this end, we adopted a structured co-development strategy in which clinical requirements were first elicited directly from movement disorder clinicians through targeted discussions conducted during and after hands-on use of the technically stable prototype configuration. These clinician interviews focused on clinical relevance, workflow integration, handling, hygiene, and potential interference with standardized neurological examinations. The results of these interviews are the clinical requirements, which led to a first clinically deployable baseline configuration that was integrated into routine inpatient motor assessments. Following each clinical session, patient usability and acceptability were systematically assessed using the System Usability Scale (SUS) [19] and short free-text comments. Clinician input was collected qualitatively through structured discussions during and after hands-on use, focusing on workflow integration, handling, hygiene, positioning, and interference with standardized neurological examinations. These data were not only used as outcome measures but also served as input for further device refinement. The predefined evaluation endpoints were acquisition stability, workflow compatibility, SUS-based usability, and redesign-relevant patient and clinician feedback.

Free-text patient comments and clinician notes were reviewed by the study team. Comments were first screened for concrete design- or workflow-relevant statements and then pragmatically grouped into recurring themes such as comfort, fit, weight distribution, visual perception, appearance, hygiene, handling, and workflow compatibility. These groupings were used descriptively to identify recurrent usability barriers and redesign priorities.

### 2.5. Participants

The study was conducted at the Department of Neurology of the University Medical Center Hamburg-Eppendorf (UKE), Germany. At this center, people with PD are routinely admitted for approximately two weeks for diagnostic workup and optimization of dopaminergic medication and, where applicable, programming of deep brain stimulation systems.

Early during admission, all patients undergo a standardized movement laboratory assessment of about three hours. In this assessment, dopaminergic medication is temporarily withheld to obtain a defined medication OFF state. The complete MDS-UPDRS part III is performed in the OFF state, a standardized dose of levodopa is administered, and the full MDS-UPDRS III is repeated in the medication ON state. This protocol allows direct comparison of motor function across OFF and ON conditions and provides a structured setting for integrating the cap-based facial recording.

The study population comprised 50 inpatients with PD who underwent routine ON/OFF assessments. Inclusion criteria were age 18 years or older, a diagnosis of PD with motor fluctuations or medication-refractory tremor, current in-patient treatment at the Department of Neurology at UKE, German language proficiency, and written informed consent to participate in the study. Exclusion criteria were absence of written consent, indications of a dementia syndrome, or insufficient German language skills to understand the study information and questionnaires.

### 2.6. Ethical Considerations

The study protocol is approved by the Ethics Committee of the Ärztekammer Hamburg (reference number: 2024-101380-BO-ff) and is conducted in accordance with the principles of the Declaration of Helsinki. All participants receive written and oral information about the purpose, procedures, and potential risks of the study and provide written informed consent prior to participation, including consent for video-based recording of their face, storage of the recordings, and linkage to relevant clinical data.

To protect privacy and data security, the system operates over a closed, internal Wi-Fi connection between the cap and the receiver. All recordings are stored locally in a structured SQLite database using pseudonymized patient identifiers. Privacy considerations were implemented directly at the software level, including restricted display of recordings to the indexed patient and optional background blurring to minimize incidental capture of third parties. Access to the app and recordings is restricted to authorized clinical and research staff.

### 2.7. Statistical Analysis

A mixed-effects linear regression was employed to analyze the data. The dependent variable was the SUS score. The fixed-effect predictor was a binary variable indicating device version, coded as 0 for the original prototype and 1 for the redesigned prototype. The model was fitted using the full set of available SUS observations from both prototype evaluations, rather than the paired subset alone. A participant-level random intercept was included to account for the paired subset of 15 participants who evaluated both prototype versions. Prototype 4 was additionally evaluated in additional participants, resulting in 50 observations for the final prototype. Residual diagnostics, including a QQ plot comparing sampled and theoretical quantiles and Levene’s test for homogeneity of variance, were inspected to assess model assumptions.

## 3. Results

The wearable hypomimia system was refined against three interrelated requirement domains: (1) engineering requirements ensuring technical feasibility and stable data acquisition, (2) clinician-derived workflow requirements enabling integration into routine neurological assessments, and (3) patient-derived comfort and acceptability requirements enabling sustained wear during multi-hour ON/OFF evaluations. These requirement domains were addressed sequentially but iteratively. Technical verification constituted the prerequisite baseline for clinical deployment, which in turn enabled systematic usability evaluation and patient-centered redesign.

### 3.1. Verification of Technical Requirements

Pre-clinical bench testing demonstrated that the clinically deployable baseline configuration fulfilled all predefined engineering acceptance criteria. Continuous dry-run recordings showed a mean battery endurance of approximately 4 h and 15 min under streaming conditions. No excessive heat generation was observed, with device temperature remaining stable throughout prolonged operation. Wireless data transmission via the closed Wi-Fi connection was highly stable when the device and receiver were located in the same room, with no connection losses recorded. In addition, the integrated camera module operated reliably across repeated recordings, and the 3D-printed housing proved mechanically robust, supporting safe handling and wear during use. These tests were used to identify and resolve technical failure modes prior to clinician interviews.

### 3.2. Overview of Iterative Co-Development and Evaluation

**Figure 2:**
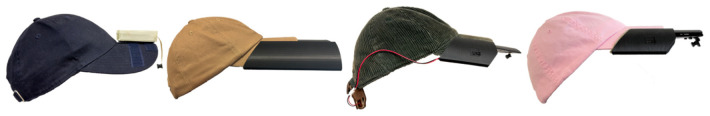
This paper describes four distinct prototypes. The development stages (Prototype 1 to 4) are shown from left to right. Prototype 1 and 2 were solely technically evaluated, while Prototype 3 and 4 underwent clinical refinement.

Following the establishment of a technical proof-of-concept in the pre-clinical development phase, we report the empirical findings that guided the subsequent co-development of the wearable hypomimia capture system (Figure 2). First, we report clinician-derived clinical requirements elicited through structured interviews and hands-on use of the technically stable prototype configuration. These requirements informed the development of the first clinically deployable baseline (Prototype 3). Second, we present patient-reported usability outcomes and qualitative feedback obtained during routine ON/OFF motor assessments, which guided the refinement of the system into the final prototype (Prototype 4). Finally, quantitative usability outcomes based on the SUS are reported to evaluate improvements across prototype versions.

### 3.3. Clinical Requirements

To inform the transition from technical proof-of-concept to routine clinical use, movement disorder clinicians were systematically consulted during and after hands-on use of the technically stable prototype configuration. These targeted discussions focused on clinical relevance, workflow integration, handling, hygiene, and potential interference with standardized neurological examinations. Pragmatic grouping of clinician feedback yielded a set of recurring clinical requirements that defined the acceptance criteria for the first clinically deployable prototype.

A central requirement concerned compatibility with established clinical workflows. Clinicians emphasized that the wearable system must integrate seamlessly into the standardized ON/OFF motor assessment without prolonging examination time or distracting from core neurological tasks. Donning and adjustment of the cap needed to be fast, intuitive, and feasible without technical assistance. The device was required to remain stable throughout the multi-hour assessment without repeated readjustment.

Closely related, clinicians highlighted the importance of unobstructed neurological examination. The camera housing and brim geometry were required not to obscure the patient’s eyes, mouth, or facial musculature, nor interfere with visual contact during MDS-UPDRS item 3.2 (facial expression) ratings.

Patient safety and comfort emerged as further core requirements. Clinicians stressed that the device must not generate noticeable heat, exert excessive pressure on the forehead, or introduce instability during head movements. Weight distribution was identified as particularly critical for longer assessments, as frontal load could lead to discomfort or slippage over time.

From a clinical operations perspective, hygiene and reusability were identified as non-negotiable requirements. The wearable system had to be easily disinfected between patients and compatible with hospital hygiene protocols. Clinicians explicitly preferred solutions that avoided permanently integrated electronics in textiles and instead supported modular components that could be cleaned, replaced, or upgraded independently.

Finally, clinicians emphasized the need for data reliability and transparency during acquisition. A live video preview was considered essential to verify correct camera positioning and full-face coverage prior to recording and to avoid unusable datasets. Stable wireless connectivity and reliable data capture throughout the assessment were regarded as prerequisites for any future clinical or research use of the system.

Taken together, these clinician-derived requirements defined the functional and practical criteria for routine clinical deployment.

### 3.4. Prototype 3

#### 3.4.1. Resulting Clinically Deployable Prototype

Based on the clinician-derived requirements, the wearable system was redesigned into a first clinically deployable baseline configuration (Prototype 3). This iteration focused on aligning the technically validated concept with routine clinical workflows, hygiene standards, and extended wear during standardized ON/OFF motor assessments.

To support seamless integration into clinical workflows, the device architecture was consolidated into a self-contained, modular unit that could be mounted and adjusted quickly without technical assistance. The cap-based carrier remained unchanged, but the electronics housing was restructured to allow stable positioning without repeated readjustment during prolonged assessments. Clinicians were able to don and align the device prior to the examination using live video preview, after which no further interaction with the system was required during routine neurological testing.

In response to requirements regarding unobstructed examination and visual contact, the geometry of the hardcase and brim extension was refined to ensure consistent full-face capture while preserving direct line of sight between clinician and patient. The camera position was fixed relative to the brim to reliably capture both upper and lower facial regions within a single frame without interfering with facial inspection or MDS-UPDRS item 3.2 ratings.

To address comfort and safety during extended wear, the internal component layout was redesigned to reduce frontal weight and improve balance across the cap. Heat generation and pressure points at the forehead were minimized through component redistribution and enclosure shaping. These changes aimed to support continuous wear throughout the multi-hour ON/OFF assessment without causing discomfort or instability.

Consistent with clinician feedback on hygiene and reusability, the modular separation between textile carrier and electronics was preserved and further emphasized. The hardcase containing all electronic components could be removed independently from the cap, allowing standard disinfection procedures between patients and flexible use of different caps without duplicating electronics.

Stable wireless streaming and live video preview ensured reliable and transparent data acquisition, allowing clinicians to verify correct alignment and full-face coverage prior to recording and reducing the risk of unusable datasets. Together, these properties established Prototype 3 as the first configuration suitable for routine inpatient deployment, where it was integrated into standardized ON/OFF motor assessments and used as the baseline for systematic usability evaluation and subsequent patient-centered refinement.

#### 3.4.2. Quantitative Usability Outcomes

Prototype 3 represented the first clinically deployable baseline configuration and was therefore evaluated with patients during routine motor assessments. The usability was assessed using the SUS following completion of the standardized assessment protocol. Based on fifteen observations, Prototype 3 achieved a mean SUS score of 44.7 (SD 18.2), reflecting moderate usability accompanied by considerable interindividual variability. The participants had a mean age of 62.2 years (SD 6.6; range 51.0–74.8 years) and included 13 male and 2 female individuals. The mean disease duration since diagnosis was 9.2 years (SD 5.3). These scores served as the quantitative baseline for later comparison following patient-centered refinement.

#### 3.4.3. Qualitative Patient Feedback

To complement the SUS ratings and to identify concrete design-related challenges that should be addressed for the next prototype, patients were invited to provide short free-text comments after wearing Prototype 3. Pragmatic grouping of free-text feedback revealed recurring themes related to fit, comfort, visual perception, and appearance.

The most frequently reported issues concerned fit and wearing comfort. Several patients described the cap as sitting “too steep” on the head or exerting pressure on the forehead, particularly in individuals with smaller head circumferences or when worn over glasses. In some cases, the cap was reported to slip laterally during longer assessments. At the same time, patients whose textile cap size matched well explicitly reported high wearing comfort, indicating sensitivity of comfort to fit rather than general intolerance of the concept. Across comments, weight and weight distribution emerged as central factors. Heavier brim configurations were described as increasingly noticeable over time and perceived as tiring during the multi-hour assessment, even when initial placement was acceptable.

Patients also commented on visual perception and camera placement. Some reported that the extended brim or camera module entered their field of view, cast shadows, or restricted vision in certain positions. From an esthetic perspective, the visible wiring and external battery components of Prototype 3 were described as conspicuous and “technical,” which several patients felt would limit acceptability beyond a purely laboratory setting.

These findings directly informed the subsequent patient-centered redesign of the device into the final prototype version.

### 3.5. Prototype 4

#### 3.5.1. Patient-Centered Redesign

Based on the patient-reported usability outcomes and qualitative feedback obtained from evaluation of Prototype 3, the wearable system was further refined into a final prototype configuration (Prototype 4). This redesign focused on addressing patient-identified limitations related to comfort, weight distribution, visual perception, and appearance, while preserving the clinically validated functionality established in Prototype 3.

To improve wearing comfort and fit, the internal layout of electronic components was reorganized to reduce frontal load and achieve a more balanced weight distribution across the cap. The overall mass of the brim-mounted module was reduced through component miniaturization and enclosure redesign, with the aim of minimizing pressure on the forehead during prolonged wear.

**Figure 3:**
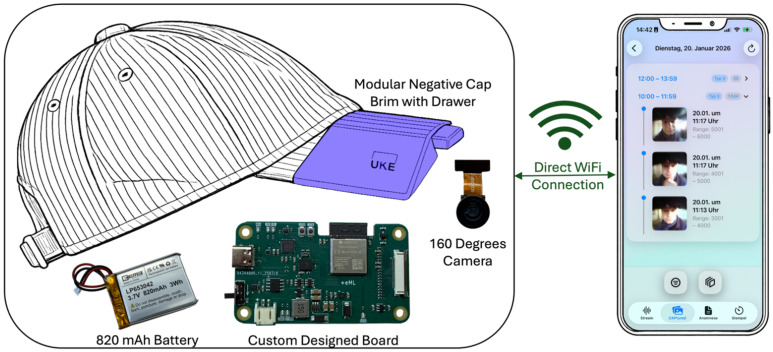
The architecture of our final Prototype 4 and its connectivity to any iOS device.

In response to feedback concerning visual intrusion, the camera housing and connecting bridge were shortened and more closely integrated into the brim geometry. This reduced the extent to which the device entered the wearer’s peripheral visual field and limited shadowing effects reported with the previous configuration. To address concerns regarding appearance and everyday acceptability, visible external wiring and auxiliary components were eliminated. Electronics were fully integrated into the brim-mounted hardcase, resulting in a more compact and visually cohesive design. Transparent and illuminated elements were reduced or concealed to minimize technical salience (Figure 3).

Importantly, these refinements did not alter the core functional characteristics of the system. Full-face capture, live video preview, stable wireless transmission, and modular separation of textile and electronics were retained to ensure continued compatibility with clinical workflows and hygiene requirements.

#### 3.5.2. Quantitative Usability Assessment

Following implementation of the patient-centered redesign, Prototype 4 was evaluated using the same usability assessment procedures applied to Prototype 3.

Based on 50 observations, the mean SUS score for Prototype 4 was 80.0 (SD 12.6), indicating good overall usability. Participants had a mean age of 62.4 years (SD 8.1; range 38.6–78.9 years). The cohort included 31 male and 19 female participants, and the mean disease duration since diagnosis was 9.8 years (SD 4.3). Of the 15 participants who evaluated Prototype 3, all also evaluated Prototype 4, providing a paired subset for direct within-participant comparison.

**Figure 4:**
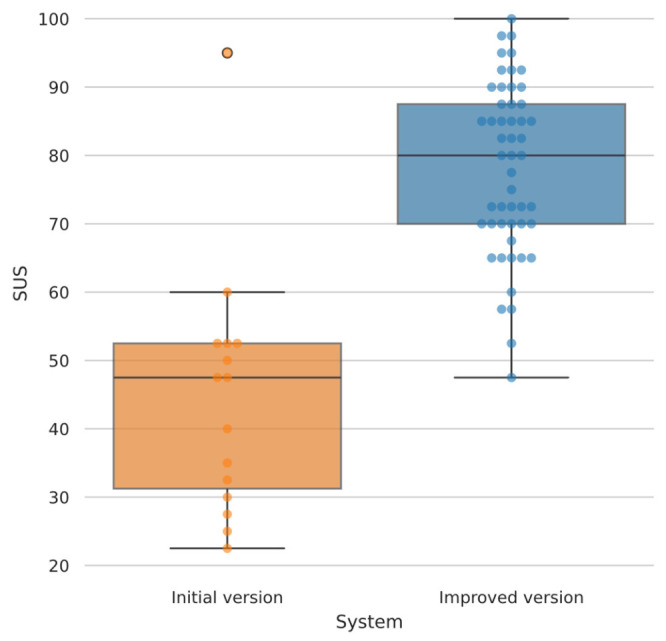
The SUS results from our clinical-used prototypes: Prototype 3 (initial version) was assessed on 15 patients and Prototype 4 (improved version) on all 50.

A mixed-effects linear regression model demonstrated a statistically significant improvement in usability compared with Prototype 3 (Figure 4). The model yielded an intercept of 44.5 (±3.0), representing the expected SUS score for Prototype 3, and a coefficient of 33.6 (±3.0) for Prototype 4. The redesigned device achieved, on average, a 33-point higher SUS score. The 95% confidence interval for this difference ranged from 27.7 to 39.4 (*p* < 0.001). Residual diagnostics showed no evidence of heterogeneity of variance based on Levene’s test (*p* = 0.301), while the Shapiro–Wilk test indicated deviation from residual normality (W = 0.952, *p* = 0.012). Given the small sample size, the straightforward interpretability of the untransformed coefficients, the robustness of linear mixed-effects models to mild normality violations, and the model’s primary use as a descriptive measure, we deemed this potential deviation acceptable.

#### 3.5.3. Qualitative Patient Feedback

Qualitative feedback obtained after use of Prototype 4 reflected many of the changes introduced in response to earlier patient comments. Patients frequently described the redesigned device as more comfortable and less noticeable during wear. The reduced weight and improved balance were explicitly mentioned as improvements, particularly during longer assessments.

Comments related to visual perception indicated fewer reports of visual obstruction or shadowing compared with Prototype 3. From an esthetic perspective, patients described the redesigned cap as more discreet and visually acceptable, with fewer technical elements drawing attention. A small number of patients still noted the presence of the frontal camera bridge, suggesting potential for further miniaturization in future iterations.

Overall, patient feedback confirmed that the redesign addressed key limitations identified during evaluation of Prototype 3. Patients also explicitly recognized improvements relative to the earlier configuration, indicating that their feedback had been meaningfully incorporated into the device design.

#### 3.5.4. Data Acquisition

**Figure 5:**
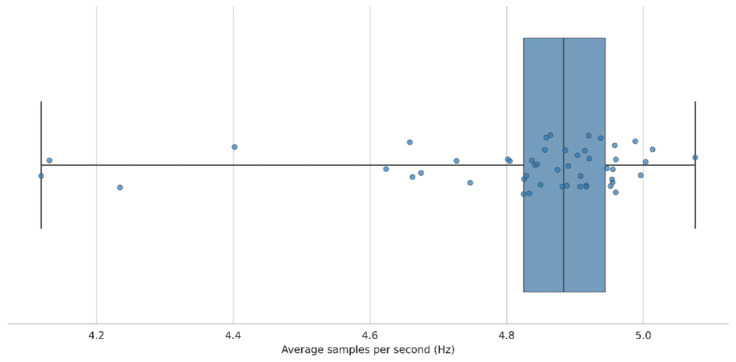
The sampling rate of the final prototype for all 50 patients.

Although technical verification (Table 1) was addressed prior to usability analyses, quantitative acquisition metrics are reported here to demonstrate how the system performed during actual clinical deployment across all recorded patient sessions. Across 50 patients, the system achieved a mean effective image sampling rate of 4.79 frames/s (SD 0.33). The median sampling rate was 4.89 frames/s, with an interquartile range from 4.81 to 4.92 frames/s. Maximum observed rates approached 4.98 frames/s, while isolated lower values (minimum 2.99 frames/s) reflected short event windows rather than sustained acquisition failure. Importantly, no systematic frame loss or progressive degradation over time was observed (Figure 5).

In absolute terms, individual assessment segments comprised several hundred to more than one thousand image frames per session (median ≈ 760 frames). Across all samples, faces were successfully detected in 95.4% of frames (1,386,641 of 1,453,652 total frames), with a mean per-patient detection rate of 94.6%, indicating robust and consistent performance across participants. Taken together, these findings confirm that the system reliably fulfilled its predefined technical acquisition requirements in real-world use, thereby establishing a robust technical baseline for the subsequent clinician- and patient-centered usability evaluation.

## 4. Discussion/Conclusions

This work shows that a wearable approach to hypomimia capture can be brought into routine inpatient motor assessments when technical feasibility, clinical workflow constraints, and patient experience are treated as equally important design inputs rather than sequential afterthoughts. Although many digital hypomimia approaches demonstrate promising performance from short, stationary recordings, the practical barrier for translation is often sustained wear and unobtrusive integration into examination routines. The present results suggest that this barrier can be addressed through iterative co-development that combines early clinician grounding with later patient-centered refinement. Importantly, the approach used here should not be understood as a replacement for established user-centered design methodologies, but as a clinically staged application of such principles to wearable monitoring systems. Its distinguishing feature is the separation of technical readiness, workflow readiness, and patient wearability as distinct but interdependent development steps.

A first point that emerges is the role of “good-enough” acquisition as a prerequisite for meaningful usability iteration. The stable sampling behavior observed during routine ON/OFF assessments indicates that a conservative acquisition strategy can provide sufficiently dense datasets while avoiding common wearable failure modes such as heat generation, processing overload, and unstable wireless transmission. In this setting, technical stability is not the final outcome but an enabling condition: it prevents data loss and reduces operational friction, allowing clinicians and patients to focus their feedback on comfort, handling, and acceptance rather than on basic functionality. The observed sampling rate should therefore be interpreted in relation to the intended level of analysis. The MDS-UPDRS Part III motor examination is an observation-based clinical assessment lasting approximately 20 min, and previous wearable-sensor work has emphasized that motor states should be evaluated over clinically meaningful assessment windows rather than isolated snapshots [20]. At approximately 4.8 frames/s, the present system provides repeated facial samples across such windows and is therefore suitable for future analyses that compare spatial facial configuration, frame-wise expressivity scores, or distributional landmark features between medication states [21]. Accordingly, occasional frame loss is expected to affect derivative-based temporal features more strongly than window-level summary measures, which will be the primary target of subsequent clinical validation studies.

At the same time, the usability findings make clear that technical stability does not imply usability. Prototype 3, despite meeting acquisition requirements and fitting the workflow, was rated only moderately usable and showed large interindividual variability. This gap is important because it reflects aspects that are difficult to anticipate in preclinical testing. The subsequent increase in SUS scores after redesign, together with consistent qualitative improvements, suggests that the main usability barriers were not subtle preferences but concrete ergonomic factors that accumulate during multi-hour wear. Patient comments repeatedly pointed to frontal load, pressure on the forehead, fit, and intrusion into the visual field. These aspects are rarely visible in short trials, yet they strongly determine whether a wearable device remains tolerable across an entire assessment and whether it would be acceptable beyond a purely experimental context.

Clinician and patient involvement contributed in different ways and at different times. Clinicians primarily shaped feasibility within clinical routines, emphasizing rapid donning, reliable positioning, hygiene compatibility, and the need for live video preview to prevent unusable datasets. Patients, in contrast, articulated the practical limits of sustained wear and the social-esthetic dimension of acceptability, including the perception of the device as conspicuous or “technical.” Treating these perspectives separately, rather than aggregating them into a single notion of “user feedback,” helped identify which barriers were workflow-related and which were embodied and experience-related. This staged separation is a transferable element of the framework: for other wearable monitoring systems, clinicians may be best positioned to define clinical utility, workflow compatibility, and data-quality safeguards, whereas patients are essential for identifying comfort, appearance, burden, and sustained-use barriers. The usability improvement between prototypes supports the idea that patient involvement is not only an ethical requirement but a direct driver of measurable usability gains when feedback leads to concrete redesign decisions.

Rapid prototyping enabled this process in a practical way. The use of 3D printing and a modular hardcase architecture within a university medical center made it possible to implement geometry and component-layout changes quickly, test them under real conditions, and iterate without the overhead of conventional manufacturing. Modularity also supported hygiene requirements by separating textile and electronics, allowing disinfection routines and component exchange without redesigning the full system. In this context, rapid prototyping was less a technical detail and more a development strategy that allowed iterative feedback to translate into tangible improvements within a clinically realistic timeline. Although the specific device was developed for facial monitoring in PD, this development logic may be applicable to other wearable systems that require stable acquisition during clinical routines, such as movement, gait, posture, tremor, dyskinesia, or other video- or sensor-based monitoring tools.

Several limitations define the scope of interpretation and next steps. First, paired usability observations were limited to 15 participants who evaluated both prototype versions in a non-randomized sequence, even though the observed improvement was large and statistically robust. Second, usability was evaluated in a single inpatient setting, and longer-term use should be assessed to determine whether comfort and acceptance remain stable over repeated wear and in less controlled environments. Usability in shorter and less standardized assessments should also be examined, even though prolonged ON/OFF assessments likely represent a conservative test condition for wear-related burden. Third, despite improvements, the system remains a visible and still relatively heavy wearable device, and further miniaturization will likely be needed to improve everyday acceptability and reduce reports of visual intrusion. Fourth, the current approach depends on a nearby phone for streaming and storage. Thus, increasing autonomy through secure on-device storage or buffered recording (for example, on an SD card) would reduce operational dependence and support broader deployment. Finally, this study focused on device development, feasibility, workflow integration, and usability, and did not evaluate clinical validity. Quantitative facial feature extraction, comparison with established clinical ratings such as the MDS-UPDRS item for facial expression (3.2), and assessment of responsiveness across medication states are therefore required in subsequent validation studies.

In summary, the findings support the position that successful translation of wearable hypomimia monitoring depends on aligning technical design choices with both clinical workflow constraints and patient experience. The iterative co-development approach used here resulted in a technically stable system with substantially improved usability, providing a strong basis for subsequent studies on clinical validity and real-world utility.

## Figures and Tables

**Table 1:** The technical specifications of all four prototypes.

Specification	Prototype 1	Prototype 2	Prototype 3	Prototype 4
Feedback from	Clinicians		Clinicians and patients	
Hardcase	3D-printed rectangular box on top of brim	3D-printed negative cap brim with hollow core		3D-printed modular negative cap brim with drawer
Weight without cap (g)	38	161	180	127
Dimensions l × w × h (cm)	7 × 3.5 × 2 (box)	13 × 15.5 × 1.7 (hardware unit)	13 × 7.5 × 2 (hardware web)	11.5 × 8 × 2 (hardware web)
Board	Waveshare ESP32 One	Custom-designed V1	Custom-designed V2 (smaller)	
Board Software	Factory software with custom C++	Custom C++		
Processor	ESP32-D0WDQ6-V3	ESP32-S3-MINI-1-N8		
Camera	OV2640 21 mm 66 degrees	OV2640 21 mm 120 degrees		OV2640 21 mm 160 degrees night vision
IMU	none	Bosch BNO055		
Storage	Micro-SD	none	Stream to iOS device	
Battery	none		2000 mAh	820 mAh
Connector	Micro-USB	USB-C		
Hygiene compliance	no	yes		Improved by modularity
Accompanied iOS software	none		Rust backend and Swift frontend	

## Data Availability

The source code underlying this study is publicly available in the Research Data Repository of the University of Hamburg under the DOI: 10.25592/uhhfdm.18590 (accessed on 20 April 2026).
